# Development of a neonatal curriculum for medical students in Zimbabwe – a cross sectional survey

**DOI:** 10.1186/s12909-018-1194-2

**Published:** 2018-05-02

**Authors:** Simbarashe Chimhuya, Bothwell Mbuwayesango, Eva M. Aagaard, Kusum J. Nathoo

**Affiliations:** 1 Department of Pediatrics and Child Health, University of Zimbabwe College of Health Sciences, P.O.Box A178, Mazoe Street, Avondale, Harare, Zimbabwe; 2 Department of Surgery, University of Zimbabwe College of Health Sciences, Harare, Zimbabwe; 3 Department of Medicine, Division of General Internal Medicine at the University of Colorado School of Medicine, Aurora, CO USA

**Keywords:** Curriculum, Medical education, Undergraduate, Neonatal, Newborn

## Abstract

**Background:**

Calls have been made to reassess the curricula of medical schools throughout the world to adopt competence-based programs that address the healthcare needs of society. Zimbabwe is a country characterized by a high neonatal mortality rate of 24 per 1000 live births. The current research sought to determine the content and appropriate teaching strategies needed to guide the development of an undergraduate neonatal curriculum map for medical students at the University of Zimbabwe College of Health Sciences.

**Methods:**

We surveyed faculty (*n* = 8) and non-faculty pediatricians (*n* = 5), senior resident medical officers (*N* = 26) using a self-administered questionnaire, and completed one focus group discussion with midwives (*n* = 11). We asked respondents their expectations regarding knowledge, psychomotor skills, competencies, and teaching strategies in a basic newborn curriculum for medical students. Relevant policy and curricula documents were reviewed to assess newborn health needs and the current training. A group of faculty educationists (*n* = 11) collated and finalized the findings from the document review, survey, and focus group using descriptive statistics and thematic analysis.

**Results:**

The document review revealed three key neonatal health objectives according to the current national maternal and neonatal health road map. These objectives are to be met using a four tier approach comprising (i) family planning (ii) focused antenatal care (iii) clean and safe delivery and (iv) basic and comprehensive emergency obstetric & neonatal care. Existing curriculum has 15 newborn topics taught in lecture style during the pediatric rotations, and five newborn care skills to be learned through observation. The existing curriculum is silent on desired competencies. In the current study 19 cognitive areas, 17 psychomotor skills and six competency domains were identified for an ideal neonatal curriculum for undergraduate students. A combination of teaching strategies including classroom, simulation and a clinical rotation were recommended.

**Conclusion:**

This study revealed a significant gap between the existing neonatal curriculum and the ideal curriculum as recommended by broad stakeholders in the context of national health care needs. Next steps are to complete the development and implementation of the proposed curriculum map to better align with the ideal state.

**Electronic supplementary material:**

The online version of this article (10.1186/s12909-018-1194-2) contains supplementary material, which is available to authorized users.

## Background

By 2015, the global burden of neonatal mortality was estimated to be 2.9 million, constituting 44% of the total under-five mortality. Predominantly these deaths occurred in low and middle-income countries including India (779,000 deaths), Nigeria (276,000 deaths), Pakistan (202,400 deaths), China (157,400 deaths), and Democratic Republic of Congo (118,100 deaths), which contributed more than 50% of the burden [1]. Sub-Saharan Africa and Southern Asia have both the worst mortality burden and achieved the slowest reduction in mortality rates between 1990 and 2015.

The major causes of newborn deaths worldwide are preterm complications, intrapartum events, and infection-related illness. Low-cost interventions are available that can reduce these causes of neonatal mortality by 58, 79, and 84%, respectively [2]. These include investing in care during labor, through the first week of life, improving the maternal and newborn quality of care and reducing inequities in care by reaching out to every woman and newborn. On this background, the Every Newborn Action Plan (ENAP) was created as a global roadmap and renewed commitment to end preventable newborn deaths and stillbirths by 2035 [3].

Zimbabwe is among the countries that, despite a reduction in under-five mortality rate from 102 (1999) to 75 (2015) failed to achieve its Millennium Development Goal (MDG) target of 34 deaths per 1000 live births per year [4]. The neonatal mortality rate remained unchanged at 29 per 1000 live births during the same period. A national integrated health facility assessment (NIHFA) report on the state of healthcare facilities and quality of service in health institutions in Zimbabwe highlighted significant knowledge and skills gaps among health workers in managing common newborn conditions [5]. The transfer of neonatal skills to healthcare workers must begin in the undergraduate period and continues throughout the career of healthcare professionals [6–9].

In response, the Zimbabwe Ministry of Health and Child Care (MOHCC) has been implementing a number of in-service training programs targeting nurses, midwives, and doctors at the primary and secondary care level. These programs include, among others, Helping Babies Breath, Kangaroo Care, Baby Friendly Hospital Initiative (BFHI), Community Based Distributor (family planning), Basic Emergency Obstetric and Neonatal Care (BEmONC), Comprehensive Emergency Obstetric and Neonatal Care (CEmONC), and Prevention of Mother To Child Transmission (PMTCT) of HIV. The question that arises is how medical schools can also contribute to the initiatives through strengthening the pre-service curriculum in order to produce cadres of doctors with the relevant skills to provide essential newborn care.

The University of Zimbabwe – College of Health Sciences (UZCHS) is the largest and longest standing medical school in the country. The undergraduate medical training is divided into two phases, the preclinical (years 1 and 2) and the clinical (years 3-5) curriculum. This is followed by another 2 years of housemanship (internship) during which time the new graduand, now designated ‘junior doctor’ rotates through each of the departments of medicine, surgery, pediatrics, obstetrics/gynecology, psychiatry, and anesthetics. After completing the rotations and being internally certified by each of the departments, the doctor may be deployed to other hospitals. This doctor is expected to diagnose common conditions, provide appropriate treatment, and make reasonable judgments to refer complicated cases whilst providing lifesaving care during the process of referral.

There has been a call for medical training reform within the college so that graduands are better aligned with the health needs of the country. The award of a Medical Education Partnership Initiative (MEPI) Grant to UZCHS by the US government in 2010 was pivotal in driving new medical education initiatives at the college. Partner institutions on this grant included University of Colorado School of Medicine (UCSOM), University of Colorado Denver Evaluation Center (UCDEC), and Stanford University. With collaboration from the US institutions, the Novel Education Clinical Trainees and Researchers (NECTAR) program was launched at UZCHS [10]. The NECTAR program sought to improve faculty through medical education courses, research support, and strengthening of both undergraduate and postgraduate courses. A major objective of NECTAR was curricular review and development. This work was formally commenced in 2013. Part of the preparatory activities for this work involved identifying and adopting desired competencies for college graduands. These are: 1) medical expert 2) ethical professional 3) scholar 4) communicator/relationship builder 5) community health advocate 6) educator and 7) manager/leader. These competencies were based on the CanMeds framework [11]. Similar initiatives have been adopted elsewhere [12, 13].

## Methods

### Context

In the existing undergraduate curriculum at UZCHS, newborn medicine is taught as a component of pediatrics. In this needs assessment we intended to develop a curriculum map [14] for a newborn curriculum for undergraduate medical students that may be used to prepare the students to deliver newborn health care aligned to the needs of the country. Curriculum mapping has been adopted in many settings as a useful tool to align curriculum to some required standards. It is a continuous process of reviewing the needs (of leaners, teachers and society), scope, teaching styles, learner evaluations, learning environment as well as timetables. We sought to determine the current knowledge content and clinical skills being taught on basic newborn care, the ideal curriculum as defined by local health needs and input from experts, as well as appropriate and feasible strategies to teach the curriculum.

### Design

Cross sectional study.

### Participants

The participants in the survey were faculty and non faculty pediatricians, Provincial Medical Directors of all the nine provinces of the country, senior resident medical officers in pediatrics, and midwives working at one of the teaching hospitals.

### Data collection methods

We employed mixed methods to conduct the needs assessment [15]. These included reviewing relevant curricula and policy documents, survey of medical educators and recent granduands from the medical school, as well as holding focus group discussion with midwives.

#### Document review

We reviewed key documents to guide curriculum review on newborn care including the existing (1995 version) UZCHS undergraduate curriculum working document for MBChB degree. We examined this document to establish the current content of the newborn curriculum and associated teaching strategies. We accessed the Zimbabwe Maternal and Neonatal Health Road Map 2007-2015 [16] document for national objectives and strategies to address newborn healthcare needs. The latter document was reviewed to establish the goal state as well as identify the critical priority areas for our country.

#### Survey

We developed a structured questionnaire and distributed it to pediatricians and senior resident medical officers in pediatrics (for detail about the questionnaire see Additional file 1). The questionnaire sought to identify stakeholder perceptions regarding the knowledge, psychomotor skills, and competencies required in a basic newborn care curriculum for medical students. We did not validate the questionnaire. On knowledge and psychomotor skills, we asked respondents to list five priority conditions or health problems of the newborn and five high priority skills they recommend to be taught to undergraduate medical students.

Respondents were also asked to tick all that applied from seven listed competency domains adopted by the UZCHS. They were not asked to assign a ranking of the competencies. We also asked the respondents to suggest ideal strategies for teaching these competencies most effectively. In this survey, we did not gather views about methods of student assessment because we felt it was not ideal timing to include this since various departments of the college including pediatrics were piloting other methods of learner assessments such as OSCE and standardized patients. In addition, we felt assessment strategy should be determined based on the level of the learning objectives. This will be part of next steps in the completion of the curriculum map.

The questionnaire was distributed to pediatric faculty (*n* = 13) via group email. Nonfaculty received the questionnaire by email distributed by the Paediatric Association of Zimbabwe, a professional organization of Zimbabwe pediatricians. Over 90% of pediatricians in the country were members of this association (*n* = 28) at the time of the survey. Eleven pediatricians who responded in the survey also attended a subsequent group discussion to finalize the curriculum. The questionnaire was delivered physically to all junior doctors (*n* = 30) in the department of pediatrics at Harare Central Hospital through the office of the administrative secretary. Harare Central Hospital is a tertiary level referral center. It is one of the two teaching hospitals for the College of Health Sciences where undergraduate and postgraduate students receive training in neonatal care and other disciplines.

#### Focus group discussion

One focus group discussion was held with midwives working in the neonatal unit and maternity at Harare Central Hospital (*n* = 11). It was more convenient to gather the midwives into a group. The structured questionnaire was used to guide discussion, gather information and ultimately come to consensus on their opinions regarding each of the questions. Consensus was sought amongst midwifes in order to focus the discussion on key issues for the curriculum from their perspective. This mimicked the survey where we also limited responses to a certain number. One researcher mediated the discussion and guided the debate until a consensus was reached. The second researcher jotted down key notes and the final consensus statement of the group. The consensus was verified with the group at the time of the discussion.

### Analysis

Survey data were analyzed in Excel using descriptive statistics. For open ended questions and the focus group, we used inductive analysis whereby the themes emerged from the survey data. The process involved researchers sitting together and reading successive survey responses searching for meanings, recognizable patterns or ideas in the responses of participants [17]. A list of topic codes was drawn. Similar topics or items expressing the same overall idea were clustered together into the final categories or themes. Disagreements were resolved through discussion.

## Results

### Document review

Through review of the existing curriculum for undergraduate medical training at the UZCHS, we identified the neonatology topics administered by the department of pediatrics during the 4th and 5th years of training. These topics are shown in Table 1 below. Lectures on neonatal surgery were recently added and delivered through the department of surgery. The main teaching strategy was didactic lectures delivered to blocks of students attached to pediatrics. Five newborn care skills to be learned through observation were listed in the students’ logbook. The current curriculum is silent on desired competencies.

**Table 1:** Neonatology topics and skills taught during 4th and 5th years according to the existing curriculum

*Knowledge area*	*Knowledge area*	*Skills*	*Teaching strategy*
*4th-year Neonatal Tutorials: one week block teaching* Normal newborn/assessment of gestational age Resuscitation of the newborn Thermoregulation Low-birth weight (IUGR/prematurity) Neonatal seizures Neonatal sepsis Neonatal jaundice Congenital infections Hypoxic ischemic encephalopathy (HIE) Respiratory Distress in newborn X-rays – general surgical condition Metabolic conditions Fluid & electrolytes Congenital malformations Feeding the newborn	*5th-year Neonatology Tutorials:* one week block teaching Resuscitation, APGAR scores, Asphyxia Birth trauma Hypothermia Neonatal jaundice Low birth-weight Hypoglycemia Respiratory problems (including Hyaline Membrane Disease) Infections and immunity Antibiotics in the neonate Convulsions Meningitis Anemia Bleeding problems Normal hematological values in neonates Vomiting Intestinal obstruction Common surgical problems	peripheral vein puncture technique, lumbar puncture, endotracheal intubation, cardiopulmonary (newborn) resuscitation rapid glucose estimation (use of glucometer)	*Classroom* *Observe newborn procedural skills*

The second key document reviewed was the Zimbabwe Maternal and Newborn Health Road Map 2007-2015. The three road map objectives that relate to newborn care are:

1. To increase the availability and utilization of quality focused antenatal care including HIV Prevention of Mother to Child Transmission (PMTCT) services:
2. To improve access to skilled attendance at delivery including emergency obstetric and newborn care;
3. To improve access to quality postnatal care including PMTCT services

To meet these objectives the roadmap provides a framework that builds on four pillars. These pillars are (i) addressing the family planning needs of women as a strategy to improve maternal health, reduce low birth weight rate, reduce preterm birth rate and reduce overall neonatal deaths; (ii) promote a focused antenatal care which prescribes a minimum 4 antenatal consultations during a pregnancy with specific activities spelt out for each consultation. These activities are aimed at identifying high risk pregnancies, diagnosis and treatment, and birth preparedness; (iii) clean and safe deliveries attended by skilled and adequately equipped health care providers; and (iv) provision of basic emergency obstetric & neonatal care (BEmONC) and comprehensive emergency obstetric & neonatal care (CEmONC).

### Survey

**Fig. 1:**
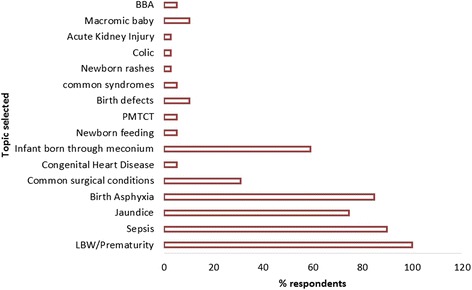
Topics selected by pediatricians and senior resident medical officers to teach under the proposed newborn curriculum in Zimbabwe. These are broad topics or content areas that were selected by pediatricians and senior resident medical officer. BBA (born before arrival - infant that is born outside a health institution)

Thirteen of 28 (46.4%) pediatricians returned completed questionnaires within 4 weeks of receiving it. Eight of the respondents were members of faculty representing 73% faculty responders whereas the other five represented 31% of non-faculty members. Of the 30 senior resident medical officers (SRMOs) rotating through Harare Central Hospital, 26 completed and returned the questionnaire (86.7%). At the time of the survey, 14 of the responders were in the middle of the pediatric rotation whereas the other 12 were completing. Figure 1 below shows the recommended content areas for the newborn curriculum in Zimbabwe as identified by pediatricians and senior resident medical officers.

**Fig. 2:**
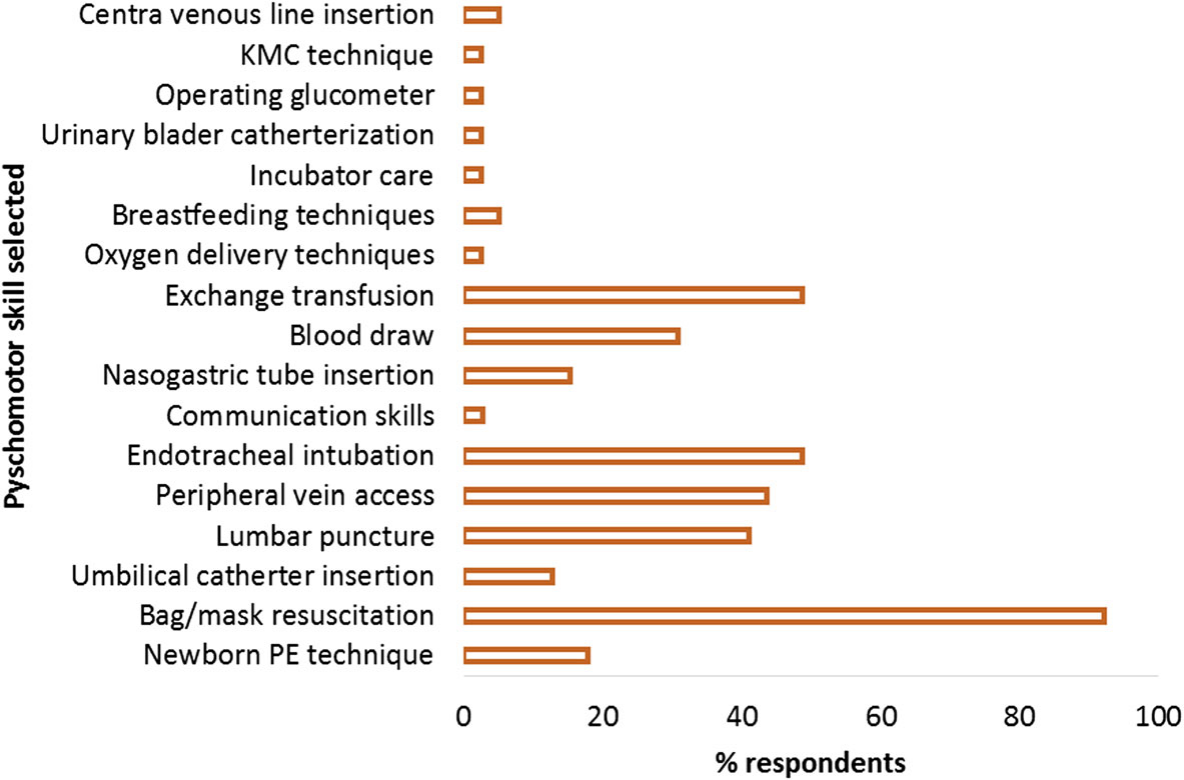
Psychomotor skills selected by pediatricians and senior resident medical officers to teach under the proposed newborn curriculum in Zimbabwe. In this figure we present the manipulative skills and techniques that the respondents in the survey regarded as crucial to train medical students. The type of skills are shown on the X-axis and frequency of selection by respondents the on the Y-axis. KMC (kangaroo mother care), PE (physical examination)

Figure 2 below shows the psychomotor skills identified by pediatricians and senior resident medical officers for the proposed newborn curriculum.

Six of the seven competency domains were selected by pediatricians. These are medical expert (100%), community health advocate (46.1%), ethical professional (46.1%), communicator/relationship builder (46.1%), manager/leader (46.1) and educator (15.4%).

#### Teaching methods

Combinations of methods of instruction were recommended in order to effectively deliver the newborn curriculum. The most commonly recommended methods were clinical rotation on the neonatal unit and classroom teaching. During the proposed clinical attachment, students were expected to obtain patient medical history, do formal case presentations during bedside teaching, observe and perform procedural skills.

### Focus group discussion

The focus group discussion of midwives revealed certain priorities of newborn care their observation of inadequate skills among newly graduated doctors as they rotate through the neonatal intensive care unit. The findings are shown in Table 2 below.

**Table 2:** Knowledge, skills, and competencies suggested by midwives to teach under the proposed newborn curriculum in Zimbabwe

Competencies	Knowledge	Practical skills
° Medical expert ° Community health advocate ° Communicator and relationship builder ° Ethical professional ° Manager/leader	Infection control and prevention – knowledge of spread of infection on nursery, through direct contact from patient to patient in close proximity, disinfection of hands and equipment Occupational safety – safe disposal of sharp objects and dirty/used items, self-protection against injury or injury to others Protection of babies against cold exposure – during clerking, bedside procedures, resuscitation, and physical examination	Insertion of peripheral line – the newly qualified doctors show a need for more training Resuscitation of newborn babies in labor ward – performing bag/mask resuscitation correctly Endotracheal intubation – technique of placing the tube and securing it

The results from the document review, survey and focus group of midwives were put together in a basic curriculum map as shown in Table 3 below.

**Table 3:** A basic map for UZCHS neonatal curriculum for medical students

Competencies	Cognitive/knowledge areas	Psychomotor skills	Teaching strategies
Medical expert Community health advocate Ethical professional Communicator/ relationship builder Manager/leader Educator	1. Prevention of mother to child transmission of HIV (PMTCT). Students familiarize with national PMTCT guidelines 2. Pathophysiology, causes and management of birth asphyxia 3. Understand the care of infant born through meconium 4. Understand the care of baby born before arrival 5. Infection a. Diagnose and manage sepsis b. Understand principles of prevention and control 6. Understand principles of protection of babies against cold exposure 7. Problems of LBW/prematurity 8. Kangaroo mother care 9. Understand principles of newborn feeding and calculate nutritional requirements 10. Principles of occupational safety 11. Pathophysiology and management of neonatal jaundice 12. Common newborn surgical conditions (gastroschisis, omphalocoel, eosophageal defects) 13. Review of common newborn rashes 14. Review of common congenital heart diseases 15. Review common birth defects (cleft lip/palate) 16. Review common syndromes (trisomy 21, 18) 17. Pathophysiology of acute kidney injury 18. Review of causes and management of macrosomia 19. Management of colic	Perform these procedures 1. Perform Bag/mask resuscitation 2. Perform Newborn examination systematically 3. Administer Oxygen using different techniques (nasal prongs, face mask, headbox) 4. Perform Blood draw from a peripheral vein 5. Place and secure a baby in KMC position 6. Assist mother to Breastfeed a baby 7. Insert and secure Nasogastric tube 8. Measure blood sugar using glucometer 9. Insert and secure a Peripheral vein line 10. Clean an Incubator after use 11. Communicate medical information to a mother Observe the following procedures 12. Endotracheal intubation 13. Lumbar puncture 14. Exchange transfusion 15. Umbilical catheter insertion 16. Urinary bladder catheterization 17. Central venous line insertion	Classroom teaching Clinical attachment on a neonatal unit Skills training laboratory

## Discussion

This needs assessment was a vital step in the development of a curriculum map for a basic newborn curriculum for undergraduate medical students at UZ-CHS. This framework highlights 19 knowledge areas, 17 critical psychomotor skills and 6 competency domains of importance in the training of medical students on newborn care. It also highlights key gaps in the current curriculum and suggests potential mechanisms by which to teach these areas. We will discuss key findings of interest with regard to competency domains, priority conditions, and teaching methods identified as critical gaps to address in order to achieve our educational goals.

The physician survey results placed the highest emphasis on producing medical experts and community health advocates for newborn health as the paramount competencies. The curriculum should, therefore, inculcate these competencies in the medical students. In addition to the routine duty of diagnosing and treating diseases, physicians should also be prepared to provide advocacy for community health and work at governance level to influence policies that advance health. They should also have sound orientation toward ethical practice and possess skills of team building among all health workers. Participants in this study considered being scholarly to be least valuable to undergraduates compared to the other competencies. Importantly, the physicians’ views were similar to the midwives.

**Fig. 3:**
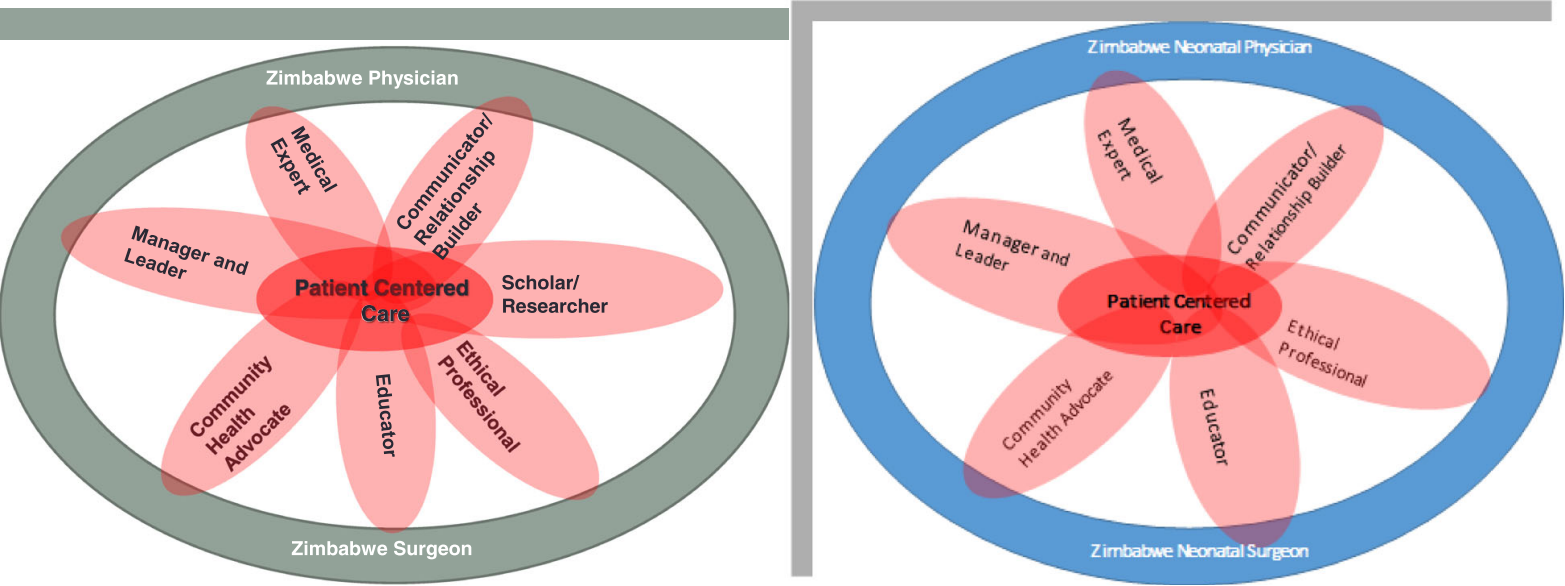
The complete Zimbabwe trained physician/surgeon (left) as envisioned by UZCHS in general and the competent neonatal physician (right) as espoused by the survey. The picture on the left shows the seven competencies adopted by the UZCHS. Each competency is shown as a petal of flower. The flower is a befitting reflection of that ideal graduand of UZCHS. On the right is the authors’ reconstruction of the flower with the six key competencies that were identified by this survey

These results are consistent with HE Jeffery’s view who argues that the desired goal of neonatal teaching should be to make students achieve sound competence on basic care skills that reflect the healthcare system of the society [6]. The attainment of higher level skills should be deferred until the postgraduate years. Thus of the seven competencies the undergraduate neonatal curriculum should strive to achieve 6 of these i.e. medical experts, community health advocate, communicator/relationship builder, ethical professional, manager/leader, and educator. This is represented graphically in Fig. 3 below.

With regards to content areas of importance, there was overlap with the current newborn topics as taught in pediatrics. Traditional topics such as birth asphyxia, acute infections, prematurity, newborn feeding, jaundice and others were recurrent. Some topics such as convulsions, fluid and electrolyte management, interpretation of X-rays, metabolic diseases, bleeding disorders and others were dropped in the current needs assessment. New topics were suggested including occupational safety, care of baby born through meconium stained amniotic fluid, and HIV. On this survey, the inclusion of aspects of HIV care (prevention of mother to child transmission) is logical given that Zimbabwe is one of the Southern African countries with a high burden of HIV/AIDS. Guidelines for the care of mother and infant in the setting of HIV infection keep changing as new evidence emerges and students need to familiarize themselves with current practice.

Some of the topics suggested are a reflection of physicians’ predominant experience at tertiary level practice. These topics include congenital heart diseases, cleft lip/palate and other surgical conditions. Such conditions are commonly encountered on the neonatal units at tertiary hospitals. Similarly, surgical conditions and infants with birth defects are often referred and managed at tertiary units such as Harare Central Hospital. However, baseline data on national prevalence are lacking locally.

With regards to teaching methods, a clinical rotation and a skills training laboratory featured prominently in the survey indicating a shift of focus from classroom based teaching. The recommendation for the establishment of a fully functional skills training laboratory is a noble one and in keeping with the current trend in many medical schools. The training of procedural skills using simulation manikins is becoming standard practice particularly in well-resourced settings with positive reports of satisfactory skills acquisition and acceptability by learners [18, 19]. It is also ideal in settings characterized by small size of teaching faculty. Treadwell et al. [20] reported positive results from the implementation of newborn teaching strategy to undergraduate classes. Faced with a large number of students and inadequate faculty at the University of Pretoria, a strategy using combination of CD-ROM with 12 skills domains, supplementary lectures, and practicing in a skills laboratory in addition to a neonatal rotation was implemented.

Medical education reform is a continuous process whose primary goal is to realign curricula with the needs of the healthcare system of the society [21]. Examples of curricula review can be found in limited resource settings too for example Rwanda [22] and India [23]. In both countries there was successful development and implementation of a neonatal training program aimed at general practitioners and nurses on a background of needs assessment findings of poorly standardized care and wide variation in practices from place to place. At UZCHS a stimulating example may be derived from the successful implementation and sustenance of an HIV curriculum for final year classes of medical students of 2011 and 2012 by the Department of Medicine. Faced with a depleted faculty with only 31% posts filled, the department designed and implemented a competence based HIV curriculum delivered in 10 team-based learning sessions [24]. Summative feedback indicated that over 90% of the students were more stimulated by the teaching strategy, enthused by the course material, and improved team spirit whereas perceived knowledge gain by students was at least 80%. This achievement in one of the college departments provides a benchmark for other similar initiatives such as the proposed neonatal curriculum mapping.

### Practical implications

The current UZCHS curriculum review initiative should make use of the results of the needs assessment presented here and adapt toward the finalization of a comprehensive curriculum map that effectively addresses the needs in newborn care training in a formal way. The complete curriculum could be of value in other settings whose national MNH strategies share similarity with ours.

### Limitations

The topics suggested in our proposed curriculum reflect real life experiences and expectations of the participants. All the respondents in this survey had experience working in tertiary hospital setting. Some pediatricians and senior nurses had worked in the Ministry of Health and Child Welfare and had more awareness of policy, and national priority issues in newborn health. Senior resident medical officers did not have other experience except residence in the teaching hospitals. Other key stakeholders including obstetricians, surgeons and community healthcare providers were not reached out to hence the experiences of the respondents in the survey may have restricted the breadth and depth of the issues that are critical for a newborn care curriculum. This may impact on generalizability of the findings.

Although the participation of stakeholders who were reached out in the survey was overall good, we did not receive current views from the provincial medical directors of health (representing MOHCC) on newborn health priorities for the country. In addition, the relatively small number of overall participants, particularly from non-faculty physicians, may result in a combination of response bias and limited generalizability. This limitation may result in a curriculum that does not adequately reflect the health needs of our country. However, we were able to examine the MOHCC roadmap document on maternal and newborn health (MNH) 2007-2015, and align our content with that information.

## Conclusion

The study identified significant deficits in appropriateness of content and teaching strategy in the existing newborn curriculum working document for medical students at the UZCHS. The proposed content as recommended by respondents in our survey, focus group of midwives and document review is better aligned with the vision of the MOHCC. It also has a strong advantage in that it incorporates the training of basic and critical psychomotor skills at an early stage of the medical career.

## Additional file

Additional file 1: Data collection tool. These are the four questions on the data collection tool that were used in this survey. The focus group discussion was also based on the same question in the data collection tool. (DOCX 15 kb)

## Abbreviations

HEALZ: Health education advanced leadership for Zimbabwe
MBChB: Bachelor of medicine and bachelor of surgery degrees
MEPI: Medical Education Partnership Initiative
MNH: Maternal and newborn health
MOHCC: Ministry of Health and Child Care
NECTAR: Novel education clinical training and research
NIHFA: National integrated health facility assessment
PAZ: Paediatric Association of Zimbabwe
SRMO: Senior resident medical officer (also called junior doctor)
UZCHS: University of Zimbabwe College of Health Sciences
