# Supplementary material for: Development of a neonatal curriculum for medical students in Zimbabwe – a cross sectional survey
Source: BMC Med Educ. 2018 May 2;18:90. doi: 10.1186/s12909-018-1194-2 (PMC5932895; doi:10.1186/s12909-018-1194-2)
Supplement: Supplementary file 1 — Data collection tool. These are the four questions on the data collection tool that were used in this survey. The focus group discussion was also based on the same question in the data collection tool. (DOCX 15 kb) [file 12909_2018_1194_MOESM1_ESM.docx]

Data collection tool

1. List five (5) topics/or knowledge areas that you consider as **key** for the newborn curriculum for 5th year medical students in this college
2. State five (5) psychomotor skills on newborn care that you consider as **key** to teach 5^th^ year medical students in this college?
3. What do you consider as the **key** competencies in newborn care that 5^th^ year medical students should be taught during the paediatric rotation (chose **all that apply** from the list below)? *Refer to attachment for a detailed explanation of each competence*
4. medical expert
5. ethical professional
6. scholarly
7. communicator/Relationship builder
8. community health advocate
9. educator
10. manager and leader

4. State the teaching strategy(ies) that you recommend for effective teaching of [1-3] above
